# Supplementary material for: Top-Down Proteomics Detection of Potential Salivary Biomarkers for Autoimmune Liver Diseases Classification
Source: Int J Mol Sci. 2023 Jan 4;24(2):959. doi: 10.3390/ijms24020959 (PMC9866740; doi:10.3390/ijms24020959)
Supplement: Supplementary file 1 [file ijms-24-00959-s001.zip › Supplementary Materials revised.pdf]

Table S1: UniProt-KB code, experimental and theoretical average mass values (Mav) ± standard deviations (SD), elution times of proteins and peptides analyzed, m/z values and charge of the multiply charged ions selected for XIC search in HPLC-low resolution MS and their PTMs and characterized by High resolution MS [41].

Proteins/peptides (UniProt-KB code)	El. Time (min ± 0.5)	Exper. (theor) Mav ± SD	m/z (charge) for XIC search	PTMs
**Acid Proline-Rich Proteins**				
PRP-1 2P[a] (P02810)	22.2	15515 ± 2 (15514-15515)	1293.9(+12), 1194.4(+13), 1035.3(+15), 970.7(+16), 913.6(+17)	N-Term(Gln->pyro-Glu), S_8(Phospho), S_{22}(Phospho)
PRP-1 1P [a]	22.9	15435 ± 2 (15434-15435)	1287.2(+12), 1188.3(+13), 1030.0(+15), 965.7(+16), 908.9(+17)	N-Term(Gln->pyro-Glu), S_8 or S_{22}(Phospho)
PRP-1 0P [a]	23.2	15355 ± 2 (15354-15355)	1280.5(+12), 1182.1(+13), 1024.6(+15), 960.7(+16), 904.2(+17)	N-Term(Gln->pyro-Glu)
PRP-1 3P [a]	21.6	15595 ± 2 (15594-15595)	1418.7(+11), 1300.5(+12), 1200.6(+13), 1040.6(+15), 975.7(+16)	N-Term(Gln->pyro-Glu) S_8, S_{17}, S_{22} (Phospho)
PRP-3 2P (P02810)	22.8	11161 ± 1 (11161-11162)	1595.5(+7), 1396.2(+8), 1015.7(+11), 931.1(+12), 859.6(+13)	N-Term(Gln->pyro-Glu) S_8, S_{22} (Phospho) Fragment 1-106 of PRP-1
PRP-3 1P [a]	23.4	11081 ± 1 (11081-11082)	1584.1(+7), 1386.2(+8), 1008.4(+11), 924.5(+12), 853.4(+13)	N-Term(Gln->pyro-Glu) S_8 or S_{22} (Phospho)
PRP-3 0P [a]	23.8	11001 ± 1 (11001-11002)	1376.2(+8), 1101.2(+10), 917.8(+12) 786.8(+14)	N-Term(Gln->pyro-Glu)
PRP-3 2P [a] desR$_{106}$	22.8	11004 ± 1 (11005-11006)	1573.2(+7), 1223.8(+9), 1001.5(+11), 847.6(+13)	N-Term(Gln->pyro-Glu) S_8, S_{22} (Phospho), R_{106} removal
P-C (P02810)	15.0	4370.9 ± 0.4 (4370.8)	1457.9(+3), 1093.7(+4)	Fragment 107-150 of PRP-1
**Statherin and PB**				
Statherin (P02808)	29.2	5380.0 ± 0.5 (5379.7)	1794.2(+3), 1345.9(+4), 1076.9(+5)	S^2(Phospho); S^3(Phospho)
Statherin 1P [a]	28.9	5299.9 ± 0.5 (5299.7)	1767.6(+3), 1325.9(+4), 1060.9(+5)	S^3(Phospho)
P-B peptide (P02814)	30.0	5792.9 ± 0.5 (5792.7)	1932.0(+3), 1449.2(+4), 1159.6(+5)	N-Term(Gln->pyro-Glu)
**Histatins**				
Hst-1 (P15515)	21.9	4928.2 ± 0.5 (4928.2)	1644.1(+3), 1233.5(+4)	S^2(Phospho)
Hst-1 0P [a]	22.0	4848.2 ± 0.5 (4848.2)	1617.4(+3), 1213.5(+4)	
Hst-3 (P15516)	17.7	4062.2 ± 0.4 (4062.4)	1355.1(+3), 1016.6(+4)	
Hst-6	14.3	3192.4 ± 0.3 (3192.5)	1065.1(+3), 799.1(+4)	Fragment 1-25 of Hst-3
Hst-5	14.6	3036.5 ± 0.3 (3036.3)	1013.2(+3), 760.1(+4)	Fragment 1-24 of Hst-3
**Cystatins**				

Cystatin A (P01040)	31.8	11005.354 ± 2 (11006.5)	1001.59(+11), 1101.59(+10), 1223.94(+9), 1376.81(+8), 1573.36(+7), 1835.42(+6)	
Cystatin A acetyl[b]	33	11047.43 ± 2 (11048.5)	1005.41(+11), 1105.85(+10), 1228.61(+9), 1382.06(+8), 1579.36(+7), 1842.42(+6)	N-Term.-α-Acetylation
Cystatin A (T96→M)	29.5	11036 ± 2,. 11036.7	1840.45(+6) 1577.68(+7) 1380.59(+8) 1227.31(+9) 1104.68(+10)	
Cystatin B-SSG[c] (P04080)	32.8	11485.8 ± 2 (11486.9)	1915.5(+6), 1642.0(+7), 1436.9(+8), 1277.3(+9), 1149.7(+10), 1045.3(+11)	C3Glutathionylation
Cystatin B-SSC[d]	32.9	11299.8 ± 2 (11300.7)	1884.5(+6), 1615.4(+7), 1413.6(+8), 1256.7(+9), 1131.1(+10), 1028.6(+11)	C3Cysteinylation
Cystatin C (P01034)	35.1	13342 ± 2 (13343.1)	1483.57(+9), 1335.32(+10), 1214.02(+11), 1112.93(+12), 1027.40(+13)	2 intrachain disulfide bridges
Cystatin D-R26 des1-5 (P28325)	37.7	13517 ± 2 (13517.3)	1690.70(+8), 1502.90(+9), 1352.70 (+10), 1229.80 (+11), 1127.4 (+12), 1040.40 (+13)	N-Term(Gln->pyro-Glu) after 1-5 residue removal, 2 intrachain disulfide bridges
**Cystatins S-type**				
Cystatin S	35.3	14186 ± 2 (14185)	1774.3(+8), 1577.2(+9), 1419.6(+10), 1290.6(+11), 1183.2(+12), 1092.2(+13), 1014.3(+14)	2 intrachain disulfide bridges
Cystatin S1 (P01036)	35.3	14266 ± 2 (14265)	1784.3(+8), 1586.1(+9), 1427.6(+10), 1297.9(+11), 1189.8(+12), 1098.4(+13), 1020.0(+14)	S^3(Phospo) on cystatin S, 2 intrachain disulfide bridges
Cystatin S1 ox	35.3	14281 ± 2 (14280.7)	1786.40(+8), 1589.70 (+9), 1429.30 (+10), 1299.50 (+11), 1191.30 (+12), 1099.70 (+13)	S^3(Phospo), W^{23} oxidation, 2 intrachain disulfide bridges
Cystatin S2	35.3	14346 ± 2 (14345)	1794.3(+8), 1595.0(+9), 1435.6(+10), 1305.2(+11), 1196.5(+12), 1104.5(+13), 1025.7(+14)	S^1, S^3 (di-Phospho) on cystat S, 2 intrachain disulfide bridges
Cystatin S2 ox	35.3	14360 ± 2 (14361)	1596.64(+9), 1437.08(+10), 1306.52(+11), 1197.73(+12), 1105.68(+13)	S^1, S^3(di-Phospho) on cystati S, 2 intrachain disulfide bridge W23 oxidation
Cystatin SN (P01037)	34.6	14312 ± 2 (14313)	1790.0(+8), 1591.2(+9), 1432.2(+10), 1302.1(+11), 1193.7(+12), 1101.9(+13), 1023.3(+14)	2 intrachain disulfide bridges
Cystatin SN des 1-4				

Cystatin SN ox	34.6	14328 ± 2 (14328)	1792.30(+8), 1593.20 (+9), 1434.00 (+10), 1303.30 (+11), 1195.20 (+12), 1103.30 (+13)	2 intrachain disulfide bridges, W23oxidation
Cystatin SA (P09228)	36.8	14347 ± 2 (14346)	1794.4(+8), 1595.1(+9), 1435.7(+10), 1305.3(+11), 1196.6(+12), 1104.6(+13), 1025.8(+14)	1 intrachain disulfide bridge
**Antileukoproteinase**				
SLPI (P03973)	26.2	11702.2 ± 1 (11706)	1952.64(+6), 1673.84(+7), 1464.73(+8), 1302.10(+9)	8 intrachain disulfide bridges
**α-Defensins**				
α-defensin 1 (P59665)	23.5	3442.5 ± 2 (3442.1)	1772.03(+2), 1148.36(+3), 861.52(+4)	2 intrachain disulfide bridges
α-defensin 2 (P59665/6)	23.5	3370.4 ± 1 (3370.9)	1686.49(+2), 1124.66(+3), 843.75(+4)	2 intrachain disulfide bridges
α-defensin 3 (P59666)	23.5	3485 ± 2 (3486.1)	1744.03(+2), 1163.03(+3), 872.52(+4)	2 intrachain disulfide bridges
α-defensin 4 (P12838)	27.2	33708 ± 1 (3709.4)	1855.71(+2), 1237.48(+3), 928.36(+4)	2 intrachain disulfide bridges
**S100A proteins**				
S100A12 (P80511)	40.0	10444 ± 2 (10443.9)	1306.5(+8), 1161.4(+9), 1045.4(+10), 950.4(+11)	M1 removal
S100A7 D27 (P31151)	37.0	11367 ± 2 (11367.8)	1422.0(+8), 1264.1(+9), 1137.8(+10), 1034.4(+11)	M^{1} removal, N-Term.-α-Acetylation, D^{27} variant
S100A8(P05109)	40.4	10833 ± 2 (10834.5)	1355.3(+8), 1204.8(+9), 1084.5(+10), 985.9(+11)	
S100A9-short (P06702)	42.2	12690 ± 2 (12689.2)	1410.9(+9), 1269.9(+10), 1154.6(+11), 1058.4(+12), 977.1(+13)	N-Term.-α-Acetylation after 1-5 residue removal
S100A9-short 1P [a]	42.2	12770 ± 2 (12769.2)	1419.8(+9), 1277.9(+10), 1161.8(+11), 1065.1(+12), 983.3(+13)	N-Term.-α-Acetylation after 1-5 residue removal, T^{108} (Phospho)
S100A9-short ox	41.3	12706 ± 2 (12705.2)	1412.7(+9), 1271.5(+10), 1156.0(+11), 1059.8(+12), 978.3(+13)	N-Term.-α-Acetylation after 1-5 residue removal, M^{89} or ^{78} or ^{76} or ^{58} oxidation
S100A9-short 1P [a] ox	41.3	12786 ± 2 (12785.2)	1421.9(+9), 1279.5(+10), 1163.3(+11), 1066.4(+12), 984.5(+13)	N-Term.-α-Acetylation after 1-5 residue removal, T108 (Phospho), M^{89} or ^{78} or ^{76} or ^{58} oxidation
S100A-long SSG[c]	41.5	13459 ± 2 (13458.1)	1346.8(+10), 1224.5(+11), 1122.5(+12), 1036.3(+13), 962.3(+14)	M1 removal, N-Term.-α-Acetylation, C^{2}glutathionylation
S100A9-long SSG[c] 1P [a]	41.5	13538 ± 2 (13538.1)	1354.82(+10), 1231.75(+11), 1129.18(+12), 1042.40(+13), 968.02(+14)	M1 removal, N-Term.-α-Acetylation, C^{2}glutathionylation, T108 (Phospho)
S100A9-long SSG[c] 1P [a] ox	41.5	13555 ± 2 (13555.1)	1507.13(+9), 1356.52(+10), 1233.29(+11), 1130.60(+12), 1043.71(+13)	M^{1} removal, N-Term.-α-Acetylation, C^{2}glutathionylation, T^{108} (Phospho) M^{93} or ^{82} or ^{80} or ^{62} oxidation

S100A9-long SSC[c]				

[a]Number of phosphorylated residues is indicated as 0P, 1P or 2P.
[b]Acetyl. indicates acetylated N-terminus
[c]SSG indicates a glutathionylated cysteine residue.
[d]SSC. indicates a cysteinylated cysteine residue.

Table S2. Demographic data of HCs, AIHp and PBCp involved in the study.

**HCs**			**AIHp**			**PBCp**		
#	age	sex	#	age	sex	#	age	sex
C1	34	F	A1	70	F	P1	60	F
C2	56	F	A2	70	F	P2	51	F
C3	42	M	A3	54	F	P3	58	F
C4	48	F	A4	57	F	P4	53	F
C5	47	M	A5	55	F	P5	64	F
C6	41	F	A6	45	M	P7	42	F
C7	44	F	A7	30	F	P8	42	F
C8	50	M	A8	74	F	P9	66	F
C10	41	F	A9	45	F	P11	55	F
C11	40	F	A10	53	F	P12	55	F
C12	43	F	A11	83	F	P13	51	F
C13	63	F	A12	55	F	P14	66	F
C14	65	M	A13	60	F	P15	41	F
C16	30	F	A14	44	F	P16	70	F
C17	31	F	A15	48	F	P17	46	F
C18	47	M	A16	67	F	P18	76	F
C19	41	F	A17	42	F	P19	70	F
C20	38	F	A18	36	M	P20	64	F
C21	33	F	A19	42	M	P21	55	F
C22	46	F	A20	74	F	P22	55	F
C23	51	F	A21	69	F	P23	52	F
C24	58	M	A22	29	F	P24	70	M
C25	39	F	A23	56	F	P25	68	F
C27	34	F	A24	52	M	P26	58	F
C28	62	F	A25	43	F	P27	81	F
C29	51	F	A26	52	F	P28	63	F
C30	58	M	A27	56	F	P29	61	F
C31	64	F	A28	43	F	P30	79	F
C32	56	F	A29	57	F	P31	66	F
C33	62	F	A30	37	F	P32	63	F
C34	67	F	A31	40	F	P33	66	F
C35	61	F	A32	64	F	P34	64	F
C36	64	F	A33	74	F	P35	55	F
C37	63	F	A34	68	F	P36	63	F
C38	63	F	A35	43	F	P37	68	F
C39	62	F	A36	51	F	P38	60	F

Table S3. XIC peak areas (25th percentile, median and interquartile range) and frequencies of proteins and peptides analyzed among HCs, AIHp and PBCp. N refers to the list position of the same components in Tables 2 and S4.

**Components**		**HCs**				**AIHp**				**PBCp**			
N	*Protein/peptide*	*XIC Peak Area*				*XIC Peak Area*				*XIC Peak Area*			
		25th perc	*median*	*75th perc*	Freq/36	*25th perc*	*median*	*75th perc*	Freq/36	*25th perc*	*median*	*75th perc*	Freq/36
1	S100A12	1.00E+04	1.00E+04	5.31E+07	10	1.00E+04	1.00E+04	1.56E+08	15	1.00E+04	1.00E+04	1.00E+04	5
2	S100A8	1.00E+04	1.00E+04	1.00E+04	4	1.00E+04	1.00E+04	6.76E+07	10	1.00E+04	1.00E+04	1.00E+04	2
3	S100A7D27	1.00E+04	1.00E+04	1.78E+06	9	1.00E+04	1.00E+04	1.45E+08	16	1.00E+04	1.00E+04	1.00E+04	4
4	S100A9_s	1.00E+04	1.71E+08	3.64E+08	24	1.00E+04	1.00E+04	6.60E+08	14	1.00E+04	1.00E+04	4.75E+08	12
5	S100A9_s_ox	1.00E+04	9.73E+07	2.03E+08	23	1.00E+04	1.00E+04	1.05E+08	10	1.00E+04	1.00E+04	2.50E+07	9
6	S100A9_s_p	1.00E+04	1.00E+04	9.19E+07	10	1.00E+04	1.00E+04	1.00E+04	5	1.00E+04	1.00E+04	1.00E+04	2
7	S100A9_s_p_ox	1.00E+04	1.00E+04	1.00E+04	3	1.00E+04	1.00E+04	1.00E+04	4	1.00E+04	1.00E+04	1.00E+04	2
8	Sum_S100A9_s_and_ox	5.98E+07	2.76E+08	5.00E+08	28	1.00E+04	1.00E+04	8.01E+08	16	1.00E+04	1.00E+04	6.12E+08	13
9	Sum_S100A9_s_and_s_p	1.00E+04	2.64E+08	4.91E+08	24	1.00E+04	1.00E+04	1.04E+09	16	1.00E+04	1.00E+04	4.75E+08	12
10	Sum_S100A9_s_p_and_p_ox	1.00E+04	1.00E+04	9.19E+07	10	1.00E+04	1.00E+04	1.00E+04	5	1.00E+04	1.00E+04	1.00E+04	2
11	Sum_S100A9_s_ox_and_p_ox	1.00E+04	1.01E+08	2.03E+08	24	1.00E+04	1.00E+04	3.16E+08	11	1.00E+04	1.00E+04	2.50E+07	9
12	Sum_S100A9_s	5.98E+07	3.21E+08	6.52E+08	28	1.00E+04	1.00E+04	1.76E+09	17	1.00E+04	1.00E+04	6.32E+08	13
13	S100A9_l_g	1.00E+04	1.00E+04	2.39E+08	17	1.00E+04	1.00E+04	6.27E+08	14	1.00E+04	1.00E+04	4.82E+07	10
14	S100A9_l_g_p	1.00E+04	1.00E+04	1.00E+04	2	1.00E+04	1.00E+04	1.00E+04	4	1.00E+04	1.00E+04	1.00E+04	2
15	S100A9_l_g_ox	1.00E+04	1.00E+04	5.47E+07	12	1.00E+04	1.00E+04	1.00E+04	6	1.00E+04	1.00E+04	1.00E+04	3
16	Sum_S100A9_l_g	1.00E+04	4.62E+07	2.80E+08	19	1.00E+04	7.66E+07	1.19E+09	19	1.00E+04	1.00E+04	2.10E+08	12
17	Cystatin_A	1.26E+08	2.02E+08	3.09E+08	34	1.69E+08	3.75E+08	9.75E+08	35	1.28E+08	2.06E+08	3.77E+08	35
18	Cystatin_A_Acetyl	2.58E+07	5.40E+07	6.99E+07	29	1.00E+04	4.25E+07	1.00E+08	24	2.46E+07	4.88E+07	8.80E+07	32
19	Cystatin_A_Acetyl_T96L	1.00E+04	1.00E+04	1.42E+07	11	1.00E+04	1.00E+04	1.49E+06	9	1.00E+04	1.00E+04	1.00E+04	8
20	Sum_Cystatin_A	1.87E+08	2.88E+08	4.38E+08	34	2.11E+08	5.15E+08	1.24E+09	36	1.91E+08	2.99E+08	4.69E+08	35
21	Cystatin_B_s_glut	3.01E+07	5.73E+07	9.57E+07	27	6.52E+06	5.00E+07	1.69E+08	27	8.41E+06	4.32E+07	1.13E+08	29
22	Cystatin_B_s_cyst	1.00E+04	1.00E+04	1.36E+07	11	1.00E+04	1.00E+04	2.92E+07	12	1.00E+04	1.00E+04	4.50E+07	14
23	Cystatin_B_S_Sdimer	1.00E+04	1.00E+04	6.12E+06	9	1.00E+04	1.00E+04	1.96E+07	12	1.00E+04	1.00E+04	1.00E+04	4
24	Cystatin_B_s_CMC	1.00E+04	1.00E+04	1.00E+04	7	1.00E+04	1.00E+04	1.00E+04	3	1.00E+04	1.00E+04	1.00E+04	2
25	Sum_Cystatin_B	4.74E+07	8.50E+07	1.74E+08	29	9.15E+06	5.45E+07	3.06E+08	29	1.11E+07	5.08E+07	1.83E+08	29
26	Cystatin_C	1.00E+04	1.00E+04	8.15E+07	14	1.00E+04	1.00E+04	1.00E+04	6	1.00E+04	1.00E+04	1.00E+04	3

27	Cystatin_D_des_1_5	1.00E+04	8.21E+07	1.62E+08	24	1.00E+04	1.00E+04	1.60E+08	13	1.00E+04	1.00E+04	1.23E+08	11
28	Cystatin_S	4.68E+04	4.83E+07	1.00E+08	27	1.00E+04	8.81E+07	2.09E+08	22	1.00E+04	1.00E+04	5.61E+08	15
29	Cystatin_S1	3.23E+08	8.16E+08	1.60E+09	32	6.55E+08	1.33E+09	2.40E+09	32	9.74E+08	4.66E+09	7.44E+09	31
30	Cystatin_S2	7.48E+07	2.21E+08	4.81E+08	30	1.74E+08	3.99E+08	1.03E+09	28	6.33E+08	2.00E+09	3.32E+09	30
31	Cystatin_SN	6.55E+08	1.45E+09	2.86E+09	33	1.09E+09	2.12E+09	4.60E+09	30	1.01E+09	5.23E+09	1.23E+10	33
32	Cystatin_SN_des_1_4	1.00E+04	1.00E+04	1.14E+08	16	1.00E+04	1.39E+08	5.93E+08	21	1.00E+04	4.67E+07	2.03E+08	20
33	Cystatin_SA	1.00E+04	2.74E+07	4.01E+08	18	1.00E+04	1.00E+04	5.82E+08	17	1.00E+04	1.00E+04	4.57E+08	15
34	Cystatin_S1_ox	1.00E+04	1.00E+04	9.68E+07	14	1.00E+04	2.24E+08	7.21E+08	25	1.00E+04	1.00E+04	5.43E+08	13
35	Cystatin_S2_ox	1.00E+04	1.00E+04	1.00E+04	3	1.00E+04	1.00E+04	2.14E+07	9	1.00E+04	1.00E+04	1.00E+04	6
36	Cystatin_SN_ox	1.00E+04	1.00E+04	1.94E+08	16	1.00E+04	1.31E+08	5.64E+08	19	1.00E+04	9.25E+07	3.74E+08	21
37	Sum_Cystatin_S1	3.64E+08	1.04E+09	1.61E+09	32	5.26E+08	1.08E+09	2.40E+09	32	1.60E+09	5.77E+09	8.15E+09	31
38	Sum_Cystatin_S2	7.48E+07	2.21E+08	5.21E+08	30	1.89E+08	3.99E+08	1.12E+09	28	6.33E+08	2.05E+09	3.48E+09	31
39	Sum_Cystatin_S_S1_S2	4.91E+08	1.52E+09	2.22E+09	32	6.56E+08	1.58E+09	3.86E+09	32	3.73E+09	8.40E+09	1.17E+10	34
40	Sum_Cystatin_SN	9.27E+08	1.72E+09	3.38E+09	34	1.29E+09	2.72E+09	5.11E+09	30	1.29E+09	5.55E+09	1.25E+10	34
41	Sum_Cystatin_SA	1.00E+03	3.96E+07	5.58E+08	36	1.00E+04	1.00E+04	5.82E+08	17	1.00E+04	1.00E+04	4.57E+08	15
42	Hst_1	1.00E+04	1.48E+08	5.47E+08	24	1.09E+08	3.54E+08	8.73E+08	29	1.28E+08	2.78E+08	5.03E+08	29
43	Hst_1_0P	1.00E+04	1.00E+04	1.00E+04	6	1.00E+04	1.00E+04	5.57E+06	9	1.00E+04	1.00E+04	1.00E+04	5
44	Sum_Hst_1	1.00E+04	1.48E+08	5.65E+08	24	1.15E+08	3.99E+08	8.83E+08	29	1.28E+08	2.87E+08	5.03E+08	29
45	Hst_6	1.00E+04	5.43E+07	1.63E+08	21	5.24E+07	1.94E+08	4.82E+08	29	1.00E+04	1.07E+08	2.12E+08	25
46	Hst_5	8.40E+07	1.75E+08	5.18E+08	28	1.78E+08	4.62E+08	1.39E+09	30	7.78E+07	3.00E+08	4.53E+08	30
47	Hst_3	1.00E+04	1.00E+04	1.45E+08	14	1.00E+04	1.23E+08	5.45E+08	22	1.00E+04	1.00E+04	1.04E+07	9
48	Sum_Hst_3	1.18E+08	2.35E+08	8.43E+08	29	3.37E+08	8.96E+08	2.32E+09	30	1.05E+08	4.71E+08	7.39E+08	30
49	Sum_Hst	1.30E+08	4.55E+08	1.40E+09	32	6.17E+08	1.48E+09	3.49E+09	30	3.66E+08	7.71E+08	1.19E+09	31
50	α_defensin_1	9.55E+07	2.83E+08	5.64E+08	35	9.58E+07	2.55E+08	7.33E+08	34	1.12E+08	3.12E+08	5.89E+08	34
51	α_defensin_2	5.95E+07	1.72E+08	4.27E+08	35	6.74E+07	1.95E+08	5.21E+08	30	7.11E+07	1.92E+08	3.38E+08	32
52	α_defensin_3	1.00E+04	6.63E+07	2.06E+08	25	1.00E+04	5.79E+07	2.85E+08	21	1.29E+07	7.19E+07	2.49E+08	28
53	α_defensin_4	1.00E+04	4.58E+07	1.08E+08	21	1.00E+04	6.18E+07	1.47E+08	23	1.00E+04	1.76E+07	8.91E+07	18
54	Sum_α_defensins	1.97E+08	6.14E+08	1.30E+09	35	1.97E+08	6.94E+08	1.73E+09	34	2.48E+08	6.33E+08	1.26E+09	35
55	PRP1_2P	4.80E+09	8.92E+09	1.37E+10	35	6.18E+09	9.56E+09	1.66E+10	36	3.20E+09	6.42E+09	1.04E+10	36
56	PRP1_1P	6.79E+08	1.22E+09	1.71E+09	34	4.40E+08	8.09E+08	1.89E+09	35	4.35E+08	7.13E+08	9.39E+08	36
57	PRP1_0P	1.00E+04	1.00E+04	8.23E+07	16	1.00E+04	1.00E+04	9.33E+07	17	1.00E+03	1.00E+03	7.07E+07	36
58	PRP1_3P	1.00E+04	1.00E+04	1.43E+08	14	1.00E+04	1.00E+04	1.83E+08	17	6.40E+07	1.36E+08	2.60E+08	28

59	Sum_PRP1	5.28E+09	1.10E+10	1.76E+10	35	6.91E+09	1.10E+10	1.99E+10	36	3.74E+09	7.24E+09	1.15E+10	36
60	PRP3_2P	1.16E+09	2.39E+09	4.30E+09	34	1.23E+09	2.86E+09	4.96E+09	34	1.30E+09	2.15E+09	3.21E+09	36
61	PRP3_1P	2.06E+08	4.52E+08	7.14E+08	33	2.31E+08	4.92E+08	8.36E+08	32	1.27E+08	2.85E+08	3.95E+08	35
62	PRP3_0P	1.00E+04	1.00E+04	2.44E+08	10	1.00E+04	1.00E+04	3.64E+08	13	1.00E+04	9.63E+05	1.86E+07	18
63	PRP_3_diphos_Des_Arg106	1.00E+04	3.22E+08	9.42E+08	23	1.00E+04	6.87E+07	8.52E+08	19	2.27E+08	4.54E+08	1.00E+09	36
64	Sum_PRP3	2.09E+09	3.76E+09	5.75E+09	36	2.79E+09	3.88E+09	6.32E+09	36	1.96E+09	2.95E+09	4.23E+09	36
65	P_C_peptide	9.74E+08	1.71E+09	2.64E+09	36	1.30E+09	2.44E+09	4.31E+09	36	1.43E+09	2.03E+09	3.10E+09	35
66	Statherin_2P	4.66E+08	1.17E+09	2.27E+09	32	1.14E+09	2.23E+09	3.84E+09	35	5.98E+08	2.10E+09	3.22E+09	36
67	Statherin_1P	1.77E+07	3.09E+07	5.49E+07	28	2.86E+07	4.83E+07	1.03E+08	29	1.00E+04	3.40E+07	5.12E+07	26
68	Statherin_0P	1.00E+04	1.00E+04	1.00E+04	4	1.00E+04	1.00E+04	1.00E+04	3	1.00E+04	1.00E+04	1.00E+04	3
69	Sum_Statherin	4.98E+08	1.18E+09	2.31E+09	32	1.18E+09	2.34E+09	3.92E+09	35	6.03E+08	2.14E+09	3.30E+09	36
70	PB_peptide	1.16E+09	2.54E+09	3.37E+09	35	1.89E+09	2.62E+09	4.61E+09	36	9.43E+08	1.53E+09	2.88E+09	36
71	SLPI	1.00E+04	1.00E+04	1.35E+07	10	1.00E+04	1.00E+04	4.81E+07	12	1.00E+04	1.37E+07	3.99E+07	23

Table S4. Scores of the Boruta algorithm for the selection of the components for RF analysis, highlighted by color tones ranging from dark to light green. N refers to the list position of the same components in Tables 2 and S3.

HCs-AIHp mixed data set			HCs-PBCp mixed data set			AIHp-PBCp mixed data set		
N	Description	Boruta score	N	Description	Boruta score	N	Description	Boruta score
34	Cystatin_S1_ox	11.1	39	Sum_Cystatin_S_S1_S2	11.7	63	PRP_3_diphos_Des_Arg106	11.4
11	Sum_S100A9_s_ox_and_p_ox	6.6	38	Sum_Cystatin_S2	9.6	30	Cystatin_S2	7.4
46	Hst_5	6.3	30	Cystatin_S2	8.9	47	Hst_3	6.8
12	Sum_S100A9_s	6.2	37	Sum_Cystatin_S1	7.4	39	Sum_Cystatin_S_S1_S2	6.5
8	Sum_S100A9_s_and_ox	6	29	Cystatin_S1	6.7	70	PB_peptide	5.4
3	S100A7D27	5.8	61	PRP3_1P	5.2	37	Sum_Cystatin_S1	5.3
48	Sum_Hst_3	5.6	27	Cystatin_D_des_1_5	4.5	62	PRP3_0P	5.1
26	Cystatin_C	5.4	63	PRP_3_diphos_Des_Arg106	4.5	16	Sum_S100A9_l_g	5
4	S100A9_s	5.2	34	Cystatin_S1_ox	4.2	28	Cystatin_S	4.4
16	Sum_S100A9_l_g	4.7	31	Cystatin_SN	4.1	46	Hst_5	4.3
45	Hst_6	4.6	28	Cystatin_S	3.9	61	PRP3_1P	4.2
49	Sum_Hst	4.3	12	Sum_S100A9_s	3.1	29	Cystatin_S1	4.1
9	Sum_S100A9_s_and_s_p	4.3	11	Sum_S100A9_s_ox_and_p_ox	2.9	13	S100A9_l_g	3.6
36	Cystatin_SN_ox	4	8	Sum_S100A9_s_and_ox	2.8	38	Sum_Cystatin_S2	2.3
5	S100A9_s_ox	3.9	62	PRP3_0P	2.7			
17	Cystatin_A	2	70	PB_peptide	2.7			
53	α_defensin_4	1.8	40	Sum_Cystatin_SN	2			
